# Redox Signaling in Diabetic Nephropathy: Hypertrophy versus Death Choices in Mesangial Cells and Podocytes

**DOI:** 10.1155/2015/604208

**Published:** 2015-09-27

**Authors:** Gina Manda, Alexandru-Ionel Checherita, Maria Victoria Comanescu, Mihail Eugen Hinescu

**Affiliations:** ^1^“Victor Babes” National Institute of Pathology, 99-101 Splaiul Independentei, 050096 Bucharest, Romania; ^2^“Carol Davila” University of Medicine and Pharmacy Bucharest, 37 Dionisie Lupu, 020022 Bucharest, Romania

## Abstract

This review emphasizes the role of oxidative stress in diabetic nephropathy, acting as trigger, modulator, and linker within the complex network of pathologic events. It highlights key molecular pathways and new hypothesis in diabetic nephropathy, related to the interferences of metabolic, oxidative, and inflammatory stresses. Main topics this review is addressing are biomarkers of oxidative stress in diabetic nephropathy, the sources of reactive oxygen species (mitochondria, NADPH-oxidases, hyperglycemia, and inflammation), and the redox-sensitive signaling networks (protein kinases, transcription factors, and epigenetic regulators). Molecular switches deciding on the renal cells fate in diabetic nephropathy are presented, such as hypertrophy *versus* death choices in mesangial cells and podocytes. Finally, the antioxidant response of renal cells in diabetic nephropathy is tackled, with emphasis on targeted therapy. An integrative approach is needed for identifying key molecular networks which control cellular responses triggered by the array of stressors in diabetic nephropathy. This will foster the discovery of reliable biomarkers for early diagnosis and prognosis, and will guide the discovery of new therapeutic approaches for personalized medicine in diabetic nephropathy.

## 1. Introduction

Diabetes is a major concern of public health, affecting more than 371 million people [1], with an expected doubling of diabetes cases by 2030 [2]. Diabetic patients might experience life-threatening macrovascular (atherosclerosis, cardiovascular disease) and microvascular complications (microangiopathy) of the retina, nervous system, and kidney [3]. Neuropathy and peripheral ischemia result in foot ulcers, often leading to amputation and severe infections [4]. All diabetes complications cause severe morbidity and raise substantial economic and societal costs.

Development of diabetic nephropathy (DN) is a major medical concern, as it greatly increases the risk of premature death by end stage renal disease and is associated with increased cardiovascular mortality. Therefore, huge research efforts are focused on deciphering pathologic molecular mechanisms in DN, which may provide valuable tools for early diagnosis and prevention of DN onset and evolution.

DN is clinically characterized by albuminuria, proteinuria, elevated creatinine levels, and abnormal glomerular filtration rates. The key pathological features of DN include glomerular hypertrophy, mesangial matrix expansion, diffuse glomerular basement membrane thickening, podocyte loss and foot process effacement, nodular glomerulosclerosis, mesangiolysis and glomerular microaneurysms, interstitial fibrosis, and tubular atrophy. Inflammation and endothelial dysfunction play important roles in DN pathogenesis. Albuminuria and afterwards proteinuria associated to glomerular changes, and interstitial fibrosis are hallmarks of DN [5].

These complex and progressive pathologic changes are mainly induced by (a) hyperglycemia and enhanced formation of advanced glycation end products (AGE); (b) increased activity of angiotensin II (Ang II) within the renin-angiotensin system; (c) excessive TGF*β*-signaling; (d) chronic inflammation associated to enhanced recruitment of leukocytes and release of proinflammatory cytokines, chemokines and growth factors [6, 7].

This review emphasizes the role of the oxidative stress in DN, which may act as trigger, modulator, and linker within the complex network of pathologic events. We highlight several molecular events underlining and connecting the metabolic, oxidative, and inflammatory stresses in DN. The aim was to bring forward key molecular networks and new hypothesis in the pathophysiology of DN, related to oxidative stress. We point out that an integrative approach is needed for identifying key molecular pathways controlling cellular responses to the complex array of pathologic signals in DN. This approach is required in order to find reliable biomarkers for early diagnosis and prognosis, and to guide the discovery of new therapeutic strategies for personalized medicine in DN.

## 2. Disease-Related Oxidative Stress in DN

Oxidative stress is linking most of the molecular events underlining the pathological process in DN, related to hyperglycemia and AGE, the renin-angiotensin system, TGF*β* signaling, and chronic inflammation. Glomerular and tubular hypertrophy, mainly due to mesangial cells accumulation, extracellular matrix deposition, thickening of glomerular and tubular basement membranes, podocyte dysfunction, and apoptosis, all are redox-induced alterations leading to albuminuria, proteinuria, glomerulosclerosis, and tubulointerstitial fibrosis.

Reactive oxygen species (ROS) are both friend and foe of aerobic organisms. They adapted to oxidative aggression by developing potent antioxidant mechanisms, and learned how to use ROS in their favor, as signaling molecules which sustain vital redox-sensitive processes. Besides phosphorylation, subtle and reversible changes of the redox status can propagate and fine-tune signals from the membrane to the nucleus.

When the tightly controlled redox balance is even slightly altered either by increased and prolonged ROS production, or by inefficient antioxidant mechanisms, pathologic processes may arise. Above a physiological limit, ROS may induce significant conformational changes of lipids, proteins, glucides and nucleic acids, leading to distorted interactions and altered cellular functions. These biologic targets practically detoxify ROS, thus interrupting the oxidative cascade. Being more stable than ROS, they are potent propagators of the deleterious action of ROS, long after ROS disappeared.

Chronic oxidative stress is a constant and ubiquitous presence in DN, accompanying and interfering with hyperglycemia and inflammation. Conventional markers of oxidative stress in serum, urine, and various organs were evidenced in DN, ranging from markers of lipid peroxidation (malondialdehyde, 4-hydroxynonenal), protein carbonyls, and oxidized DNA [8]. These few validated biomarkers of oxidative stress are insufficient for early diagnosis and prognosis in DN, and therefore huge efforts are focused on biomarker identification by deciphering the molecular basis of oxidative stress in DN and other pathologies. For instance, oxidative and glycoxidative changes of proteins, reflecting the metabolic and oxidative stresses in diabetes, are mediators of multiple distorted signaling pathways [9]. AGE are risk factors for diabetes complications, that are formed through nonenzymatic aminocarbonyl interactions between reducing sugars and oxidized lipids, proteins, amino phospholipids, or nucleic acids [10]. Oxidative stress is not only involved in AGE formation, but AGE themselves amplify oxidative stress, as described in the following sections. Hemoglobin A1c (HbA1c), a glycosylated nonpathogenic form of hemoglobin, was added to the standards of care by the American Diabetes Association, as biomarker of the presence and severity of hyperglycemia in diabetes. It exhibits less biologic variability than glucose levels and responds to diet and treatment [11]. The glycation of skin collagen and the accumulation of AGE were shown to be consistently correlated with diabetes complications. Therefore, skin fluorescence due to AGE accumulation might be considered a useful noninvasive marker of cumulative tissue damage in diabetes [12]. New AGE, like 3-dioxiglucasone, methylglyoxal, methionine sulfoxide, and 2-aminoadipic acid, were recently demonstrated to have some prognostic power regarding DN progression [13]. However, due to the sophisticated methods required for their detection, they are still far from being translated into the practice.

Several other candidate biomarkers of oxidative stress in diabetes are under study [14] and, if validated, may impact on the management of patients with diabetic complications. Moreover, the development of new therapeutic strategies definitely needs a panel of reliable, readily accessible, and easy to monitor parameters for patients follow-up.

### 2.1. Sources of ROS in DN

The ROS cascade [15] is initiated from molecular oxygen by formation of superoxide anion, either intentionally or as byproduct of other reactions. It arises from enzymatic reactions mediated by NADPH oxidases, the oxidative phosphorylation chain, xanthine oxidases, uncoupled nitric oxide synthase, and so forth. Hydrogen peroxide is then formed from superoxide anion through a reaction catalyzed by superoxide dismutases. Hydrogen peroxide may further evolve towards a highly toxic reactant, hypochlorous acid, by the action of the myeloperoxidase system. The chain involving superoxide anion and hydrogen peroxide is then continued by radical-radical reactions, to generate hydroxyl radical and singlet oxygen.

The initial steps of this cascade are under a tight enzymatic control mediated by NADPH oxidases and superoxide dismutases. (a) NADPH oxidases, having multiple components scattered in the cytosol and membranes in resting cells, are activated and translocated to the membrane for assembling a functional enzyme only when cells must respond to a particular challenge by generating superoxide anion. Numerous homologue-specific mechanisms are controlling NADPH oxidases, including calcium ions, free fatty acids, protein-protein interactions, and posttranslational modifications (phosphorylation, acetylation, or sumoylation) [16]. (b) Superoxide dismutases, are located in critical cellular compartments where superoxide anion might be generated, for avoiding unwanted oxidative damage in mitochondria and cytosol. (c) Catalase, peroxidases, and peroxiredoxins promptly detoxify hydrogen peroxide. Cells are less protected against the generation of more advanced ROS, like the hydroxyl radical, as only the availability of Fe^2+^ is limiting the Fenton reaction. Therefore, interrupting the chain of ROS generation in early phases is essential for limiting the oxidative stress damage.

#### 2.1.1. Mitochondrial ROS

A defect in the mitochondrial electron transport chain, resulting in overproduction of superoxide anion, was considered the main mechanism underlining high glucose-induced oxidative stress and subsequent DN complications [17].

*In vivo* ROS imaging evidenced that mitochondrial superoxide anion production is reduced in diabetes (the Crabtree effect) and is accompanied by diminished mitochondria biogenesis [18]. This might be an adaptive mechanism for preserving renal glomerular function during hyperglycemia-associated oxidative stress, which is mediated by AMP-activated protein kinase (AMPK), pyruvate dehydrogenase (PDH), and the peroxisome proliferator-activated receptor coactivator 1 (PGC1*α*). AMPK is the main energy sensor in the organism, normally activated for overcoming temporary caloric restriction. AMPK works in tandem with the transcriptional coactivator PGC1*α* which sustains mitochondria biogenesis and their oxidative metabolism [19]. In diabetes, AMPK is inhibited and consumes fewer electrons and oxygen, whilst carbon skeletons are released to the cytosol for use as building blocks for cell growth (cataplerosis). The reduction of carbon flow into mitochondria under conditions of caloric excess is regulated in part by the inhibition of PDH through hyperphosphorylation, leading to reduced pyruvate entry into mitochondria [20]. The decreased ROS generation in mitochondria due to low AMPK activity is sustained by concomitant PGC1*α* inhibition, leading to a limited biogenesis of mitochondria. Recovery of mitochondria (in number, structure, and function), achieved by activating AMPK with AICAR (5-aminoimidazole-4-carboxamide-1-*β*-D-ribofuranoside), was shown to restore renal podocyte function [18].

The old therapeutic strategy, aiming to interrupt mitochondrial production of superoxide anion, is questionable, as the underlining hypothesis of elevated mitochondrial ROS in diabetes might not be always true. We should be precautious with these new findings, as long as the tempo-spatial ROS alterations in diabetes are largely unknown. There is a lack of reliable* in vivo* observations, and the available experimental models have important drawbacks.

Reduced mitochondrial ROS generation in DN is uncoupled from the substantial damage of mitochondrial DNA (mtDNA), suggesting that either some other sources of ROS might be active, or non-ROS-mediated mechanisms underline DNA deletions.

Induction of DNA deletions is an early “danger response” to metabolic stress, necessary for upholding energy metabolism to rescue the cell. Above a threshold, ROS cause significant oxidative damage to mitochondria and mtDNA, and elicit apoptosis/mitophagy. Mitochondria move within the cell and frequently undergo fission and fusion. Chronic high-glucose exposure and consequent oxidative stress can induce mitochondrial morphologic changes by the activation of fission signals mediated by ERK1/2-mediated phosphorylation of dynamine-like protein-1 [21, 22]. Mitochondria fragmentation further triggers a ROS response, thus amplifying mitochondria dysfunction and cell apoptosis [23]. Fragmented and dysfunctional mitochondria are selectively targeted for degradation to autolysosomes through the multistep autophagy pathway. It is regulated by the mTOR complex-1 and AMPK sensors of the nutrient status. The elimination of damaged mitochondria is also mediated by the PTEN-induced putative protein kinase 1 and the E3 ubiquitin ligase Parkin [24]. The accumulation of fragmented mitochondria in the renal cortex in DN proves that biogenesis and clearance of mitochondria may be impaired [25, 26]. Reduced activity of AMPK, accompanied by increased signaling* via* the mTOR pathway, accounts for defective autophagy in DN. AMPK plays a central role in regulating mitochondriopathy by limiting both mitochondrial superoxide generation and autophagy of damaged mitochondria.

The evidence of reduced mitochondrial superoxide generation in DN is intriguing, considering the overwhelming evidence of advanced oxidative stress in DN. As proposed by Towler (2013), the inactivation of AMPK may shift ROS formation from the unintentional production of superoxide anion in mitochondria towards other sources, such as the Nox family of NADPH oxidases [27, 28].

#### 2.1.2. NOX Enzymes

The discovery of new members in the NADPH-oxidase family (Nox family), besides the Nox2 prototype expressed by phagocytes, which generates superoxide anion as nonspecific antimicrobial defense mechanism, changed the picture [28]. Nox family consists of 7 members, Nox1 to Nox5, Duox1, and Duox2, all of them transporting electrons across biological membranes for reducing oxygen to superoxide. They are transmembrane proteins with a certain degree of structural homology but different in their cell/tissue distribution, mechanisms of activation, and functional involvement in local redox homeostasis. The discovery of the Nox family was a breakthrough in ROS biology, emphasizing that ROS are more than toxic weapons of the innate immune response, as they are involved in redox-based signaling networks in almost all cell types [29].

The presence of various Nox isoforms, Nox4 and Nox2, was documented in renal cells and synergistically contributes to ROS generation in DN [30].

Nox4 (Renox), the most abundant Nox isoform in kidney, was found in glomeruli and proximal and distal convolute tubules. Nox4 is located in mitochondria and in the plasma membrane [31]. Nox4 exhibits a particular functional pattern among Nox members, as it is constitutively active and its contribution to oxidative stress is regulated only through expression.

Nox2 is expressed by phagocytes recruited in kidney by the macrophage chemotactic protein 1 (MCP-1) during inflammation, but also by podocytes, mesangial cells, and renal endothelial cells [31]. Unlike Nox4, Nox2 activation is tightly regulated for avoiding unintentional production of superoxide anion: its constituents are scattered in the cytoplasm and plasma membrane, and get assembled into an active membrane enzyme in response to various activation signals, through a sophisticated network of intracellular events [15].

Several studies showed that Nox4 is the main source of superoxide anion and hydrogen peroxide in DN, and that its pharmacologic inhibition or siRNA-mediated knockdown almost completely abrogates diabetic complications and the associated intracellular signaling networks [32, 33]. Inhibition of Nox4 using competitive pyrazolo pyridines inhibitors resembling NADPH may thus hold promise as therapeutic approach to prevent renal injury. As Nox4 has also a role in cardiovascular disorders [34], its pharmacologic inhibition may be a valuable tool to control DN complications more generally.

Other studies invalidated the theory stating that Nox4 is solely a major driver of renal disease; it was shown that, under particular conditions, Nox4 may limit injury and disease progression [35]. This is not surprising, knowing that Nox4 is expressed in normal renal cells in an active form, indicating that low levels of ROS, continuously produced by the constitutively active Nox4, maintain kidney homeostasis [36]. The type of experimental model and the specific experimental stressor used in these studies might explain contradictory results [37]. One may observe that Nox4 inhibitors could exert also nontargeted effects. The hypothesis that Nox4 might have a dual role, as stressor and protector of renal cells, depending on the microenvironment and the kidney pathology, has to be further explored [38].

We point out that all NADPH oxidases can act as double-edged swords triggering feedback defense against excessive ROS generation. The ROS-elicited activation of receptor tyrosine kinases and of the redox-sensitive Nrf2-Keap1 signaling pathway induces the transcription of potent antioxidants to combat the deleterious effects of chronic oxidative stress [39].

#### 2.1.3. Hyperglycemia-Induced ROS

The mechanisms of glucose metabolism involved in DN complications include glucose autooxidation, the shunt of glucose to the polyol pathway, formation of AGE, and elevated hexosamine pathway activity [40, 41].

An increased production of ROS triggered by high glucose has been suggested as a unifying process that links the pathways of hyperglycemia-induced damage: (a) the influx of glucose through the polyol pathway increases AGE formation, which, by themselves or consequent to AGE receptors signaling, cause a sustained oxidative stress and the release of inflammatory cytokines; (b) due to NADPH consumption, aldose reductase activity limits the antioxidant response to elevated ROS [40].

Although most experimental studies on DN are using high glucose as triggering stress, clinical data show that not all patients with poor glycemic control develop nephropathy, and conversely, renal complications develop sometimes even when glucose control is achieved. This evidence highlights a “metabolic memory” in diabetes, supported by a feedforward mechanism even at normal glycemia. Possibly, a vicious cycle involving AGE, oxidative stress, and epigenetic changes propagates the signals delivered initially by hyperglycemia [42].

In an oxidative environment, the polyol pathway promotes the generation of AGE, such as N-carboxymethyl lysine, N-carboxyethyl lysine, and pentosidine, and orchestrates changes leading to diabetic complications [43]. AGE propagate metabolic signals through interaction with several specific receptors, such as RAGE, macrophage scavenger receptor, and galectin-3, and induce proliferation, apoptosis, autophagy, or cell migration, depending on the target cell and the context [44]. Intracellular ROS production is triggered by AGE-RAGE interaction [45]* via* the peroxisome proliferator-activated receptor-*γ* [46]. AGE enhance the formation of cytosolic ROS which accelerate mitochondrial superoxide production. AGE-mediated signaling pathway involves functional NADPH oxidases, p21^ras^, protein kinase C, and p38 and Erk1/2 MAP kinases. Downstream transcription factors like NF*κ*B, AP-1, and SP-1 are further activated by redox-sensitive signaling pathways, triggering a plethora of pro-inflammatory and pro-fibrotic responses. AGE and even RAGE itself induce increased expression of RAGE, thus amplifying renal dysfunction. This in turn increases AGE concentrations due to reduced clearance. The cascades triggered by AGE suggest that prevention and treatment must focus not only on early glycemic control, but also on limiting the factors related to oxidative stress and AGE formation [47].

#### 2.1.4. Inflammation-Related ROS

Hyperglycemia and the associated oxidative stress promote inflammation through endothelial cell damage, increased microvascular permeability, elevated expression of chemokines, adhesion molecules, along with recruitment of inflammatory cells into the diseased kidney.

Endothelial dysfunction is an important source of oxidative stress and inflammation in DN [48]. Injured endothelial cells are active signal transducers of metabolic, hemodynamic and inflammatory factors that modify the function of the vessel wall, interact with adjacent cells, and elicit inflammatory, proliferative, and profibrotic responses. A critical mechanism of endothelial dysfunction is the impaired activity of endothelial nitric oxide synthase (eNOS), produced by posttranslational modification of the enzyme through the hexosamine pathway, S-nitrosylation, and downregulation of its expression [49]. AGE, alterations in the cellular redox state, deregulation of protein kinase C, all may contribute to eNOS impairment [50] and subsequent reduced nitric oxide (NO) bioavailability. This has important pathologic consequences, as NO opposes the effects of endothelium-derived vasoconstrictors, such as Ang II and endothelin, and protects against the damage induced by proinflammatory cytokines (TNF*α*). Oxidative stress can interfere with the production and activity of NO [50]. Superoxide anion rapidly inactivates NO and destroys tetrahydrobiopterin (BH_4_), a cofactor required for NO synthesis. At low levels of BH_4_, eNOS has been shown to generate superoxide anion instead of NO, in a NADPH-dependent manner (uncoupling of NOS activity) [51]. Increased production of superoxide anion further decreases the tissue bioavailability of NO by formation of peroxynitrite through a radical/radical reaction which takes place faster than the interaction with superoxide dismutases [52].

Neutrophils, followed by T cells, and finally macrophages are recruited from circulation, accumulate in the glomeruli and interstitium even in the early stages of DN, and inflict damage to renal and endothelial cells through cytokines, matrix degrading enzymes, and ROS [53, 54].

Neutrophils and macrophages express toll-like receptors (TLR) and consequently respond to AGE and other mediators by generating superoxide anion* via* Nox2, thus amplifying the local oxidative stress. Activated phagocytes release matrix-metalloproteinases which degrade the extracellular matrix. This in turn challenges resident and newly recruited cells to produce ROS and proinflammatory cytokines. In particular oxidative conditions, matrix-metalloproteinases might be inactivated, probably as rescue mechanism to limit oxidative stress-induced tissue destruction during inflammation [55].

CCR2-expressing monocytes are recruited in the DN kidney by MCP-1. In response to high-glucose, AGE, and oxidative stress, MCP-1 is secreted by mesangial and kidney epithelial cells, including glomerular podocytes and tubular cells. The process is mediated by NF*κ*B, at least in mesangial cells. In contrast to serum, the urine levels of MCP-1 mirror chemokine production in the kidney and correlate with disease stage and progression [56]. Pharmacologic blocking of MCP-1 seems to have beneficial effects in DN. Noxxon Pharma developed Emapticap pegol, a Spiegelmer that binds and neutralizes CCL2/MCP-1. The phase IIa proof-of-concept data showed a relevant decrease in urinary albumin excretion and better glycemic control, which persisted after treatment cessation (http://www.noxxon.com/).

In the DN kidney, blood monocytes and tissue macrophages are functionally polarized towards a proinflammatory M1 phenotype [57] and release considerable amounts of proinflammatory, profibrotic, and antiangiogenic factors (TNF*α*, IL-1, IL-6, plasminogen activator inhibitor-1, matrix metalloproteinases, TGF*β*, platelet-derived growth factor, Ang II, and endothelin) [53]. This plethora of soluble factors triggers and sustains inflammation-based pathological events in DN.

In DN, the alarmin HMGB1 (high mobility group box 1) is functioning as endogenous redox-sensitive promoter of the immune response to injury [58, 59]. HMGB1 is a nuclear nonhistone DNA-binding protein which, consequent to inflammation-induced acetylation in monocytes, translocates from the nucleus to cytoplasmic secretory lysosomes and thereafter is released [60]. Alternatively, cell necrosis, but not apoptosis, allows passive HMGB1 release from damaged or dying cells. Extracellular HMGB1 binds to various receptors, such as RAGE and TLR to which AGE also bind, and signals damage to the neighboring cells. HMGB1 is a danger associated molecular pattern [61]. Extracellular HMGB1 can act as an inflammatory mediator through MyD88, MAPK, PI3K/Akt, and NF*κ*B. Finally, HMGB1 induces recruited and resident leukocytes to release proinflammatory cytokines.

HMGB1 responds to ROS and induces ROS formation. HMGB1 is a redox-sensitive protein containing three critical cysteines which, under various oxidative settings, are oxidized or reduced and distinctively regulate HMGB1 activity. HMGB1 not only reacts to oxidative stress, but also triggers ROS generation in phagocytes by receptor-mediated activation of Nox2 [62]. The expression of HMGB1 is upregulated in the kidney of diabetic rats, suggesting that the release of hyperglycemia-triggered HMGB1 may induce renal inflammatory injury. The pathogenic role of HMGB1 is dependent on RAGE or TLR4 expression and on NF*κ*B activation, and may be related to tubulointerstitial inflammation in DN [63, 64].

The proinflammatory signals delivered by monocytes/macrophages in DN go beyond soluble factors. Recently, it was shown that communication between renal cells and monocytes/macrophages might be mediated by endothelial and monocyte-derived exosomes during inflammation. They exhibit particular signatures and may play distinctive diabetogenic and procoagulant roles [65].

### 2.2. Sensing Redox Signals in DN

In DN, renal cells are exposed and respond both to extra- and intracellular oxidative stress. Key signaling redox-sensitive pathways are either directly activated by ROS or oxidative stress-related mediators signal* via* various receptors. Pathologic consequences arise due to chronic exposure of cells to various cues and/or to transient alteration of signaling, leading to aberrant responses to stress. As multiple stressors persistently challenge renal cells in DN, it is quite difficult to differentiate between the specific pathways. Results are highly dependent on the experimental model used, including the activation system, on the stage of disease, and the time-course of the signaling events.

Cysteine is uniquely suited to sensing redox signals, as the thiol side-chain can be oxidized to several reversible redox states, such as disulphide, sulphenic acid, and S-nitrosothiol. Accordingly, proteins expressing critical cysteine residues are most susceptible to oxidant attack which modifies their structure and interaction ability by formation of disulphide bonds. This will impact on their function, including signaling and transcriptional activities. The disulphide bond status of proteins is tightly controlled by cytoplasmic and mitochondrial thioredoxins (Trx) [66] which reduce disulfides into thiol groups. The activity of Trx1 is regulated by its endogenous inhibitor, Txnip. It acts as a redox rheostat to control Trx1 activity and expression. In DN, Txnip expression is induced by high glucose conditions and Trx-mediated reduction potential is consequently limited [67].

#### 2.2.1. Protein Kinases

Various protein kinases, like PKC, Akt/B, and MAPK (ERK1/2, p38 kinase, JNK), along with phosphatases, are regulated by ROS under physiological conditions. They are also responsible for initiating and propagating disorders related to ROS overproduction [68]. We emphasize that redox and phosphorylation events are interconnected and control signaling. The consequences of these mechanisms are highly dependent on cell type, extracellular milieu, and intracellular signaling machinery, and may trigger either cell death or cell proliferation in apparently similar stressful conditions [12].

#### 2.2.2. FOXO Transcription Factors

The Forkhead box O transcription factors (FOXO1, FOXO3, and FOXO4) are redox sensors and mediators of ROS signaling in various processes and diseases, ranging from glucose metabolism to cell cycle arrest or progression, DNA damage repair, and apoptosis [69]. The oxidative stress regulates FOXO activity through posttranslational modifications (phosphorylation, acetylation, and ubiquitination). In turn, it dictates subcellular localization and transcriptional activity of FOXO. An example is the stress-activated kinase JNK which phosphorylates FOXO4, leading to its nuclear translocation and increased transcriptional activity [70]. SIRT1-mediated deacetylation of FOXO may repress its transcriptional activity, whilst Akt-dependent phosphorylation dictates ubiquitin-mediated FOXO degradation [70].

FOXO are molecular switches that decide the cell fate in response to oxidative stress, either by promoting prosurvival antioxidant responses or by triggering cell death [71]. FOXO proteins play a critical role in maintaining the redox balance. They upregulate antioxidant genes expression (manganese-superoxide dismutase and catalase) [72] or restrict mitochondrial ROS production along with mitochondria biogenesis* via* enhanced expression of heme oxygenase-1 [73]. Conversely, FOXO1 can regulate genes in both the extrinsic and intrinsic apoptotic pathways [74]. Chronic oxidative stress may induce simultaneously NF*κ*B and FOXO. Both factors have dual roles, as rescue or death-triggering factors [75]. Depending on the context, the effects exerted by each of these transcription factors and their relative balance will decide the target cell fate [76]. In certain cases, FOXO1 may act to amplify NF*κ*B-induced inflammation by binding to a response element nearby the NF*κ*B-binding element, or by physically interacting with NF*κ*B [77].

Hyperglycemia and oxidative stress alter transcription programs in target tissue cells. A “transcriptional memory” spread abnormal gene expression patterns to cell progenitors, even in the absence of the triggering stressor [78]. The existence of metabolic and transcriptional “memories” highlights the importance of early and intensive treatment in DN.

#### 2.2.3. Epigenetic Modulators

Multiple stressors trigger epigenetic changes in diabetes [79], such as DNA methylation and posttranslational modifications (lysine acetylation, methylation and ubiquitination, serine/threonine phosphorylation, and arginine methylation). Transcriptional repression or activation is dependent on the position of the residue within the histone tail and is highly regulated by paired enzymatic systems. For instance, histone lysine acetylation is mediated by histone acetyl transferases acting as transcription coactivators. Conversely, histone deacetylases (HDAC) remove acetylation marks and mostly act as corepressors. Histone-modifying enzymes can be recruited by binding to specific DNA sequences in the promoters or* via* interaction with RNA polymerase II and transcription factors [80].

HDAC can control oxidative stress* via* the transcription of Nox4; HDAC inhibition decreases Nox4 transcription in human endothelial cells by preventing the binding of transcription factors and polymerases to the Nox4 promoter, most likely due to a hyperacetylation-mediated steric inhibition [81]. In mesangial cells exposed to hyperglycemic conditions, nuclear translocation and transcriptional activation of the lysine methyltransferase Set7 are increased upon TGF*β* stimulation. This occurs downstream of ROS and is involved in glucose-driven fibrotic gene expression [82].

Sirtuins—nicotinamide adenine dinucleotide (NAD^+^)-dependent deacetylases/mono-ADP ribosyltransferases—are regulated by energy metabolism and are involved in various signaling pathways in senescence, apoptosis, DNA damage repair, and autophagy. SIRT1 exerts cytoprotection by an antiapoptotic, antioxidative, and antiinflammatory action, along with regulatory effects on mitochondrial biogenesis and autophagy [83, 84]. In stressed cells, SIRT1 shifts antiapoptotic to proautophagic responses by directly deacetylating essential autophagy proteins [85] and by deacetylation of redox-sensitive transcription factors, such as FOXO [86]. AMPK activates SIRT1 by increasing its substrate, NAD^+^ [87], but this interaction is disrupted by hyperglycemia and oxidative stress. As shown above, hyperglycemia decreases AMPK expression, leading to reduced SIRT1 expression.

Another level of epigenetic (de)regulation in DN is mediated by microRNA (miRNA). Decreased miR25 induces upregulation of Nox4 expression in mesangial cells, thus enhancing the local oxidative stress [88]. TGF*β*1 can upregulate miR192 in cultured mesangial cells and in glomeruli from diabetic mice, leading to increased collagen production by acting on specific repressors and/or by modulating other miRNA. The susceptibility of podocytes to ROS-mediated apoptosis may be partly induced by decreased miR29c levels [89].

As most molecular studies were focusing on a limited number of signaling pathways and different experimental settings, it is quite difficult to integrate existing data into a complex DN-specific signaling map. Maybe the time has come to apply an “omic” approach to integrate epigenetic changes, gene expression, and signaling in health and disease.

## 3. Hypertrophy and Death Signals in DN

Main pathological features in DN are hypertrophy of mesangial cells, glomerular extracellular matrix deposition, and podocyte loss. These different types of cellular responses to the same type of stressors reflect subtle differences in the molecular machinery in mesangial cells and podocytes.

### 3.1. Hypertrophy Responses

In DN, unopposed survival mechanisms are active, dictating mesangial cells expansion and accumulation. Mesangial cells react to high glucose, Ang II, and TGF*β* activation, by a biphasic growth response, starting with mitogen-triggered proliferation, followed by cell cycle arrest in the G1/S interphase and consequent hypertrophy. Active cell cycle-dependent hypertrophy, involving PKC activation, is initially mediated by cyclin D and is further developed by the intervention of cyclin E [90].

Mahimainathan et al. (2006) demonstrated that the hypertrophic response of mesangial cells may occur* via* the activation of the PI3K/Akt pathway due to the reduced expression and phosphatase activity of the tumor suppressor PTEN [91].

As reviewed by Brosius et al. (2010), enhanced metabolism and growth of mesangial cells could be also mediated by activation of the mTOR pathway due to reduced AMPK signaling. Activation of downstream substrates of the mTOR complex-1 triggers progrowth and antiapoptotic processes, whereas the inhibition of mTOR by rapamycin limits early glomerular hypertrophy and mesangial expansion [92].

Decreased expression and activity of the deacetylase SIRT1 favor high glucose-induced mesangial hypertrophy through downregulation of the AMPK signaling pathway and subsequent activation of mTOR [93, 94].

Unlike mesangial cells, mature podocytes do not actively synthesize DNA nor proliferate due to high levels of cyclin dependent kinases [90]. Nevertheless, podocytes might undergo ERK1/2 or Akt-mediated hypertrophic changes in DN in response to high glucose, Ang II, and to increased intraglomerular capillary pressure [95]. mTOR hyperactivation due to decreased AMPK activity in DN might also mediate a sustained hypertrophic stimulus that results in podocyte degeneration, the development of glomerulosclerosis, and proteinuria [96].

Excessive angiogenesis in DN [97] due to increased proliferation and decreased apoptosis of endothelial cells is associated with glomerular hypertrophy. Immature endothelial cells and high levels of VEGFA, which are characteristic for the early stages of DN, trigger abnormal angiogenesis and increased vascular permeability, resulting in extravasation of plasma proteins. Abnormal low levels of endothelial derived NO, along with glomerular hypertension, sustain this pathologic process. Meanwhile, in advanced stages of disease, low levels of VEGFA are registered, probably due to the inability of damaged podocytes and tubular interstitial cells to produce the angiogenic factor.

ECM accumulation contributes to glomerular hypertrophy and is considered as a repair mechanism to glomerular injury, which unfortunately escapes control in DN. ECM accumulation derives from abnormal ECM metabolism in mesangial cells and also from changes in podocytes and endothelial cells metabolism leading to the thickening of the glomerular basement membrane (GBM). Unlike mesangial cells, podocytes normally release matrix-degrading proteinases, but this control mechanism is apparently suppressed or overwhelmed in DN. Mesangium fibrosis in diabetes conditions seems to be mediated by PKC*β* stimulation of AP-1 transcriptional activity and ERK pathways, along with TGF*β*1 synthesis and signaling [98]. Additionally, JAK2 activation and phosphorylation of its downstream STAT substrates [99] mediate collagen IV and fibronectin production, TGF*β* activation, and cell growth in response to Ang II, high glucose exposure, or enhanced ROS generation [100, 101].

### 3.2. Podocyte Death

Podocytes create and preserve the glomerular perm-selectivity barrier: podocyte interdigitating foot processes are bridged by a sieve-like slit diaphragm; podocytes contribute to the synthesis of GBM; podocytes actively crosstalk with glomerular endothelia* via* VEGF and other paracrine signals. Moreover, podocyte contractile properties can modulate the hydraulic pressures sustained by the glomerulus [92].

DN is characterized by a broadening of the foot processes and podocyte loss due to cell apoptosis or detachment from the GBM. This puts a lot of pressure on remaining cells and leads to glomerulosclerosis. The morphological and functional changes of podocytes in DN are related to abnormal signaling* via* TGF*β*, MCP-1/CCR2, Wnt/*β*-catenin and VEGF [92].

Diabetic conditions increase the expression of TGF*β* and its receptor, TGF*β*RII, in glomerular cells. As reviewed by Lee [102], latent TGF*β* complexes released by mesangial cells in DN are stored in the mesangial matrix. Incompletely activated latent TGF*β* is released and localizes to the podocyte surface. In an oxidative environment, podocyte-derived plasmin, matrix-metalloproteinases, and thrombospondin-1 may activate the latent TGF*β* in podocytes. Activated TGF*β* can reduce the binding of podocytes to GBM by specific integrin downregulation, thus promoting podocyte loss [103, 104]. Activated TGF*β* delivers PI3K-mediated apoptotic signals to podocytes and subsequent activation of the proapoptotic p38-MAP kinase. This is resulting in synthesis of Bax and its translocation to mitochondria, cytochrome c release, and caspase activation, leading to apoptosis. Activation of Smad7 sustains podocyte apoptosis by inhibiting the survival factor NF*κ*B [104]. Intervention of Notch pathways in the TGF*β*-mediated apoptosis of podocytes was also suggested [105]. The activation of the apoptotic machinery by TGF*β* may also be dependent on factors controlling the cell cycle, such as the stress-induced p21 [106].

Podocyte apoptosis is aggravated by hyperglycemia and increased production of ROS and AGE, which in turn enhances FOXO4 acetylation and suppresses SIRT1 expression [107].

Autophagy is a stress response involved in the catabolic processes that degrade damaged intracellular proteins and organelles. Autophagy normally has a protective role against renal damage, but in DN it is apparently suppressed due to decreased activity of AMPK and the subsequent activation of mTOR complex-1 [26].

## 4. The Antioxidant Response in DN

The local and systemic oxidative stress underlining the pathologic features of DN is the net result of the oxidant-antioxidant balance. It may arise not only from increased ROS generation by various mechanisms, as shown above, but also from a downregulated antioxidant response. Apparently, the physiologic mechanism “ROS trigger ROS” is hyperactivated in DN, perpetuating the oxidative stress, whilst cells are unable to react properly to this overwhelming stress.

Overproduction of high glucose-induced ROS decreases* via* the PI3K–Akt–FOXO3a pathway the expression of manganese-superoxide dismutase, as guardian of superoxide generation in mitochondria [108]. Lower erythrocyte and plasma levels of reduced glutathione in type 2 diabetic patients were also registered [109]. Moreover, the sirtuin signal is downregulated and this may contribute to the failure of defense mechanisms to combat chronic oxidative stress in DN [110, 111]. This arises because Sirtuins reduce ROS formation by modulating the acetylation of the respiratory chain and by stimulating mitochondrial superoxide dismutase.

### 4.1. Dietary Antioxidants in DN

Various attempts to control oxidative stress in type 2 diabetes patients using conventional dietary antioxidants (selenium, vitamins A, C, E) failed to improve the disease outcome, as shown by the analysis performed by Akbar et al. on 14 studies [112]. The authors concluded that dietary antioxidant supplementation did not affect plasma glucose or insulin levels, but may have some benefit in protecting against the complications of type-2 diabetes. Unfortunately, no definite conclusion could be drawn due to the relatively small number of patients (572) included in these studies.

The meta-analysis performed by Bjelakovic et al. (2007) on 68 randomized trials with over 200.000 adults compared beta carotene, vitamins A, C, E, and selenium* versus* placebo or no intervention [113]. This systematic review showed that beta carotene and vitamins A and E significantly increase all-cause mortality of adults included in prevention trials. No clear evidence was found that vitamin C may increase mortality and only selenium has the tendency to reduce mortality. Biesalski et al. (2010) revised the analysis performed by Bjelakovic and concluded that dietary supplementation for the prevention or treatment of chronic diseases is most effective in those patients with inadequate intakes [114]. Apparently, there is a threshold nutrient status above which additional intake cannot provide further benefit. The threshold for nutrient intake is dependent on age, sex, health status, and gene polymorphisms.

Another approach to control oxidative stress is the adjuvant therapy with phytoantioxidants. For instance, extensive research has been focused on curcumin (diferuloylmethane), a component of the golden spice turmeric (*Curcuma longa*). In various experimental studies curcumin was shown to modulate multiple cell signaling molecules, such as proinflammatory cytokines, apoptotic proteins, transcription factors, TGF*β*, and endogenous antioxidants [115]. A small clinical study was performed on 20 patients with type 2 DN [116], showing that short-term curcumin supplementation attenuates proteinuria, TGF*β* and IL-8. Curcumin can be administered as adjuvant therapy, but long-term trials with larger numbers of patients are needed for confirmation.

### 4.2. Targeting the Endogenous Antioxidant System in DN

Advances in ROS biology and the molecular basis of the cellular response to oxidative stress indicated different ROS-associated molecules and signaling pathways as promising therapeutic targets in inflammatory disease.

Limiting ROS production may positively impact on oxidative stress-triggered pathological events, but may also negatively affect normal physiologic processes which rely on ROS. Drawbacks also arise from the multitude of ROS types produced in the respiratory burst, which makes it almost impossible to control. Therefore, a safer strategy would be to enhance the cellular response to the deleterious oxidative stress by activating the antioxidant system at transcriptional level.

The transcription factor Nrf2 (NF-E2-related factor 2), together with its negative regulator, Keap1 (Kelch-like ECH-associated protein 1), play a key role in combating oxidative stress. This is achieved by upregulating important antioxidants, like NADPH quinone oxidoreductase, glutathione S-transferase, hemeoxigenase-1, and *γ*-glutamylcysteine synthetase [117–119]* via* the antioxidant response element (ARE).

Under unstressed conditions, Keap1 represses Nrf2 transactivation activity. Keap1 appears to act as a sensor molecule of oxidative and electrophilic stresses, and it accelerates Nrf2 degradation. When Keap1 senses oxidative or electrophilic stresses, Nrf2 is liberated from Keap1-mediated repression, accumulates in the nucleus, and induces the expression of cytoprotective genes. The rapid turnover of Nrf2 prevents aberrant expression of Nrf2 target genes [120]. Nrf2 switching on and off, and Nrf2 crosstalk with signaling pathways (p53, Notch1, and NF-*κ*B) protect cells against oxidative damage, prevents apoptosis, and promotes cell survival [121]. Additionally, Nrf2 directly regulates cellular energy metabolism by modulating the availability of substrates for mitochondrial respiration [122].

Despite the chronically enhanced oxidative stress and inflammation in DN, which normally should have induced Nrf2 activation, the diseased kidney has impaired Nrf2 activity and reduced expression of its target genes [123]. Nrf2 activators, like sulphoraphane or cinnamic aldehyde, were shown to attenuate damage and preserve renal function in diabetic mice, indicating that Nrf2 might be a valuable therapeutic target in DN [124].

Nrf2 activators were designed (bardoxolone methyl and its analogues) or were extracted from broccoli (sulphoraphane). They were investigated in preclinical studies and entered clinical trials for the treatment of various diseases, like cancer and Alzheimer's disease. Both promising and disappointing results were obtained, indicating that potential side-effects due to nontargeted effects should be thoroughly considered in the first phases of clinical trials [123].

## 5. Future Perspectives

(1) We are facing a large amount of experimental information regarding the molecular interferences between oxidative, metabolic, and inflammatory stresses in DN. Most data were obtained in cell cultures and animal models. There is a need to integrate these scattered pieces into a coherent signaling map, to identify missing links, and to solve them. Major drawbacks arise from the differences between the experimental models used in various studies.

(2) Experimental studies in DN focused on small areas of the complex cellular signaling map. Despite progress, what was done until now represents only a preliminary screening, and we should restart studying DN by an “omic” approach. This may provide the “big picture” and unravel new players and connections. In this respect, solving the redox proteome will be a significant progress [125]. Nevertheless, the “omic” approach raises new obstacles, as we generate more and more data, and their integration will be even more difficult. Possibly, the “network medicine” approach may provide the models and the tools for data integration into meaningful molecular maps [126].

(3) There is no animal model of DN closely resembling human disease, especially in the late stages of DN. Therefore, extrapolation of results to humans might be inconsistent. Setup of large studies in humans is mandatory, but this is dependent on the identification of reliable and easy-to-use biomarkers of oxidative stress, which should allow monitoring of patients all along the disease course. Urinary microsomes/exosomes carry huge promise [127]. They are small vesicular structures (30–100 nm) that may be released by almost all cell types along the kidney structures, and carry a load of transmembrane, soluble, and glycophosphatidyl inositol anchored proteins, along with mRNA and miRNA. This “cargo” faithfully reflects the physiological state of the cells of origin and thus represents a reliable source of biomarkers. Moreover, exosomes participate in the cellular crosstalk which propagates signals related to metabolic, oxidative, and inflammatory stresses [65].

(4) Deciphering the molecular networks in the complex pathophysiology of DN will foster the development of new therapeutic strategies addressing either the source of oxidative stress or the cellular response to this injury. Metabolic, inflammation, and oxidative interference points should be targeted in order to concurrently address the major cues which trigger and sustain pathologic events in DN. Attacking only one of these multiple pathologic pathways is highly unlikely to be effective. Albeit the theoretical promises of such an approach, we should be aware that interfering with signaling pathways might be dangerous, sometimes unpredictable.

## Abbreviations

AGE: Advanced glycation end products
Ang II: Angiotensin II
AOPP: Advanced oxidation protein products
DN: Diabetic nephropathy
HDAC: Histone deacetylases
GBM: Glomerular basement membrane
MCP-1: Macrophage chemotactic protein-1
mtDNA: Mitochondrial DNA
PGC1*α*: Peroxisome proliferator-activated receptor coactivator 1
ROS: Reactive oxygen species
TLR: Toll-like receptors
Trx: Thioredoxins.
