# Corrigendum to “Redox Signaling in Diabetic Nephropathy: Hypertrophy versus Death Choices in Mesangial Cells and Podocytes”

**DOI:** 10.1155/2018/8515032

**Published:** 2018-11-05

**Authors:** Gina Manda, Ionel Alexandru Checherita, Maria Victoria Comanescu, Mihail Eugen Hinescu

**Affiliations:** ^1^“Victor Babes” National Institute of Pathology, 99-101 Splaiul Independentei, 050096 Bucharest, Romania; ^2^“Carol Davila” University of Medicine and Pharmacy Bucharest, 37 Dionisie Lupu, 020022 Bucharest, Romania

In the article titled “Redox Signaling in Diabetic Nephropathy: Hypertrophy versus Death Choices in Mesangial Cells and Podocytes” [1], the name of the second author was given incorrectly as Alexandru-Ionel Checherita. The author's name should have been written as Ionel Alexandru Checherita. The revised authors' list is shown above.
